# Rapid Elimination of Blood Alcohol Using Erythrocytes: Mathematical Modeling and In Vitro Study

**DOI:** 10.1155/2017/5849593

**Published:** 2017-05-31

**Authors:** Yuliya G. Alexandrovich, Elena A. Kosenko, Elena I. Sinauridze, Sergey I. Obydennyi, Igor I. Kireev, Fazoil I. Ataullakhanov, Yuriy G. Kaminsky

**Affiliations:** ^1^Laboratory of Biophysics and Physiology of the Cell, Center for Theoretical Problems of Physicochemical Pharmacology, Russian Academy of Sciences, Kosygin Street 4, Moscow 119334, Russia; ^2^Laboratory of Modeling and Bioinformatics, Institute of Theoretical and Experimental Biophysics, Russian Academy of Sciences, Institutskaya Street 3, Pyshchino, Moscow Region 142290, Russia; ^3^Laboratory of Biophysics, National Scientific and Practical Centre of Pediatric Hematology, Oncology and Immunology Named after Dmitry Rogachev, Russian Ministry of Health, Samory Mashela Street 1, GSP-7, Moscow 117198, Russia; ^4^Laboratory of Cell Hemostasis and Thrombosis, National Scientific and Practical Centre of Pediatric Hematology, Oncology and Immunology Named after Dmitry Rogachev, Russian Ministry of Health, Samory Mashela Street 1, GSP-7, Moscow 117198, Russia; ^5^Department of Electron Microscopy, Moscow State University, Belozersky Institute of Physico-Chemical Biology, Leninskie Gory 1, Building 40, Moscow 119992, Russia; ^6^Faculty of Physics, Moscow State University, Leninskie Gory 1, Building 2, Moscow 119991, Russia

## Abstract

Erythrocytes (RBCs) loaded with alcohol dehydrogenase (ADH) and aldehyde dehydrogenase (ALD) can metabolize plasma ethanol and acetaldehyde but with low efficiency. We investigated the rate-limiting factors in ethanol oxidation by these enzymes loaded into RBCs. Mathematical modeling and in vitro experiments on human RBCs loaded simultaneously with ADH and ALD (by hypoosmotic dialysis) were performed. The simulation showed that the rate of nicotinamide-adenine dinucleotide (NAD^+^) generation in RBC glycolysis, but not the activities of the loaded enzymes, is the rate-limiting step in external ethanol oxidation. The rate of oxidation could be increased if RBCs are supplemented by NAD^+^ and pyruvate. Our experimental data verified this theoretical conclusion. RBCs loaded with the complete system of ADH, ALD, NAD^+^, and pyruvate metabolized ethanol 20–40 times faster than reported in previous studies. The one-step procedure of hypoosmotic dialysis is the optimal method to encapsulate ADH and ALD in RBCs after cell recovery, encapsulation yield, osmotic resistance, and RBC-indexes. Consequently, transfusion of the RBCs loaded with the complete metabolic system, including ADH, ALD, pyruvate, and NAD^+^ in the patients with alcohol intoxication, may be a promising method for rapid detoxification of blood alcohol based on metabolism.

## 1. Introduction

Alcohol consumption has been a worldwide epidemic for centuries. According to the World Health Organization [1], the total consumption of alcohol by 7.1 billion people is 40.5 billion liters per year or, on average, 15.6 ml per person daily.

Ethanol is a domestic toxin that is voluntarily consumed by people all over the world. Alcoholism and immoderate intake of alcohol are pervasive social problems. Alcohol consumption can have a number of adverse consequences. However, an elimination of ethanol from blood is an unsolved medical puzzle, and its detoxification also remains a problem worldwide. In cases of acute alcohol poisoning, it is necessary not only to quickly reduce the concentration of ethanol in the blood, but also decrease the toxic effect of the product of its metabolism, acetaldehyde. Ethanol toxicity can be reduced biochemically using a system of two enzyme reactions: ethanol oxidation to a stronger toxin acetaldehyde catalyzed by alcohol dehydrogenase (ADH, EC. 1.1.1.1) and subsequent acetaldehyde oxidation to harmless acetate by aldehyde dehydrogenase (ALD, EC 1.2.1.5) [2]. However, the direct introduction of these enzymes into the blood in vivo will inevitably be complicated by their too rapid excretion from the body and the development of the immune response as a result of the host's reaction to a foreign protein. From this point of view, the use of erythrocytes as carriers for these enzymes is a very promising strategy.

There is a growing body of evidence supporting the successful use of carrier erythrocytes (red blood cells, RBCs) in clinical practice [3–8]. The idea was advanced four decades ago [9] and is slowly expanding in experimental and clinical applications [8, 10, 11]. Biocompatibility, minimization of the immune and allergic reactions, cell survival in circulation, and high intrinsic metabolism all make the RBC a promising bioreactor for allowing foreign proteins and enzymes to function in patients and to neutralize toxins.

Another research strategy takes advantage of the RBC as an active bioreactor rather than a passive carrier. One potential approach for reducing blood levels of alcohol and acetaldehyde involves intravenous administration of RBCs loaded with ADH and ALD [12]. However, the rates of ethanol disappearance from plasma when using enzyme-loaded RBCs were lower by an order of magnitude than those expected from the activities of encapsulated enzymes. When human erythrocytes were loaded with 13–17.8 U of ADH per ml and 0.3–0.37 U of ALD per ml (corresponding to the activities of 780–1070 and 18–22 mM/h, resp.), they removed ethanol in 30 mM solution in vitro at a rate of approximately 0.5 mM/h (calculated from the data of Lizano et al. [13]). This activity was 36 to 44 times lower than the maximal activity of encapsulated ALD, (i.e., the rate-limiting enzyme in the ethanol-metabolizing pathway) and 1560–2140 times lower than the maximal activity of encapsulated ADH. However, according to our hypothesis, the rate of ethanol clearance in vivo in the presence of erythrocytes containing encapsulated ADH and ALD is limited not by the activity of the enzymes included in the erythrocytes but by the concentrations of other metabolites participating in the reactions. Thus, the rate of ethanol clearance can be raised to levels essential for therapy with an increase in the concentrations of these metabolites. Our present study is designed to encapsulate an outward metabolic pathway into RBCs for ethanol detoxification and to identify rate-limiting factors of ethanol oxidation using these RBC bioreactors (referred to as alcocytes according to Kaminsky et al. [14]). We tested the functional deficiency of encapsulated enzymes, the lack of pyruvate availability for the lactate dehydrogenase (LDH), and a low nicotinamide-adenine dinucleotide (NAD^+^) concentration as rate-limiting factors using mathematical modeling and in vitro experiments. The obtained results showed the possibility of producing alcocytes with the therapeutically significant levels of included enzymes activity.

## 2. Materials and Methods

### 2.1. Materials

ADH, ALD, LDH, acetaldehyde, NAD^+^, adenosine triphosphate (ATP), adenine, sodium pyruvate, inosine, glutathione (GSH), glutaraldehyde, and 4-(2-hydroxyethyl)-1-piperazine-2-ethanosulphonic acid (HEPES) were obtained from Sigma (St. Louis, MO, USA). NADH was purchased from GERBU (Gaiberg, Germany). Other chemicals were of highest purity commercially available.

### 2.2. Preparation of RBC Suspensions

RBCs were isolated from stored human blood, washed twice in three volumes of phosphate-buffered saline (PBS, 10 mM NaH_2_PO_4_, containing 140 mM NaCl, pH 7.4) to remove leukocytes and platelets, and suspended in PBS to hematocrit level of 45%.

RBCs count, a mean corpuscular volume (MCV), a mean corpuscular hemoglobin content (MCH), and a mean corpuscular hemoglobin concentration (MCHC) were determined using the Hematology Analytical System (Sysmex).

### 2.3. Preparation of Alcocytes

ADH (0.8−1.3 *μ*kat/ml) and ALD (0.2−0.25 *μ*kat/ml) were added to RBC suspension. Enzymes were encapsulated using a procedure of hypoosmotic dialysis [23, 24] that was carried out as one-, two-, or three-step dialysis. All procedures were performed at 4°C.

In one-step dialysis, cell suspension was dialyzed for 45 min against 200 ml of the hypoosmotic medium consisting of 15 mM Na_2_HPO_4_, 5 mM glucose, 1.5 mM ATP, 3 mM GSH, 2 mM MgCl_2_, and 2.3 mM mercaptoethanol (pH 7.4) in a dialyzer (PSN, Baxter) with a polysynthane membrane. Sealing of RBCs was carried out by adding 0.1 volume of 33 mM Na_2_HPO_4_ buffer (pH 7.4) supplemented with 5 mM adenine, 100 mM inosine, 20 mM ATP, 100 mM glucose, 100 mM sodium pyruvate, 4 mM MgCl_2_, 200 mM NaCl, and 1.6 M KCl per one volume of dialyzed RBCs and by incubation at 37°C for 30 min. Loaded cells were washed thrice in 100 mM Na_2_HPO_4_ buffer (pH 7.4) supplemented with 6 mM glucose followed by centrifugation at 4°C (370*g* for 10 min) and removal of supernatant.

At the first step of two-step dialysis, the RBC suspension (4-5 ml) was passed through the dialyzer with the outer solution osmolality 157–166 mOsmol/kg. Then the suspension of spherical RBCs was passed through the dialyzer with the outer solution containing 100 *µ*M NAD^+^ with osmolality of 100–122 mOsmol/kg (second step). Thereafter, the loaded cells obtained in the two-step dialysis were sealed as described above.

In experiments with encapsulation of ADH and ALD by three-step dialysis, RBCs after the second step were immediately dialyzed the third time against a hypotonic solution (osmotic pressure 79–104 mOsmol/kg) containing 100 *µ*M NAD^+^. Then cells were sealed as described above for one-step dialysis.

### 2.4. Changes in Osmotic Pressure of Dialysis Solution

To investigate the dependence of encapsulation of ADH and ALD on the osmotic pressure of the dialysis solution at the second dialysis step, hypoosmotic cell swelling instead of dialysis was used at the first step of RBC treatment. Washed cells were suspended using the hypoosmotic solution (153–159 mOsmol/kg) at volumes ratio 1 : 5. These suspensions were allowed to stand at 22°C for 15 min and then centrifuged at 370*g* for 7 min. The swollen cells of the pellet were resuspended in the same hypoosmotic solution to 60% hematocrit. Then the suspensions were supplemented with ADH and ALD and divided into three equal parts. Each part was dialyzed against the outer medium with osmolality of 56–67, 88–96, or 115–118 mOsmol/kg. Then the cells were sealed as described above.

The osmotic pressure was measured with an advanced micro-osmometer apparatus (Advanced Instruments, Norwood, MA, USA).

### 2.5. Osmotic Resistance of Alcocytes

Osmotic resistance of RBCs was determined as osmolality of the solution which induced hemolysis of 50% of the cells (Wc50). According to [25] the percentage of the cells that escaped lysis at specific osmolality of the solution was determined by measuring absorbance at 650 nm in a series of cell suspensions in solutions with different osmolality. The absorbance (light scattering) in this case is determined only by the concentration of undestroyed cells in each of the solutions. A curve representing the percentage of surviving cells versus osmolality was constructed. The absorbance of the RBC suspension at an osmotic pressure of 300 mOsmol/kg was taken as absorbance at zero percent of hemolysis.

### 2.6. Microscopy

#### 2.6.1. Differential Interference Contrast Microscopy

Images of initial human erythrocytes and erythrocytes after procedure of hypotonic dialysis (in the absence or presence of ADH (50 IU/ml of RBCs suspension)) with subsequent sealing of the cells in isotonic medium were obtained by microscopy with differential interference contrast (DIC). The cells were fixed with 2.5% glutaraldehyde in PBS (pH 7.4) and images were obtained using Zeiss Cell Observer Z.1 microscopy, objective 100x 1.3 NA, camera QuantEm 512sc.

#### 2.6.2. Transmission Electron Microscopy

Transmission electron microscopy (TEM) was performed for the samples of the initial native RBCs and the RBCs after hypoosmotic dialysis and after the sealing procedure with additional 2 h relaxation of sealed alcocytes.

The washed cells were fixed overnight with freshly prepared 2.5% glutaraldehyde in PBS (pH 7.4) at 4°C. After rinsing 4 times with PBS (pH 7.4), the samples were postfixed in 1% osmium tetroxide (Ted Pella) for 1 h. Then each sample was dehydrated in a graded acetone series and embedded in Epon 812 (Sigma Aldrich, St. Louis, MO, USA). Ultrathin sections (~120 nm) were produced using Ultracut E (Reichert, Vienna, Austria). The sections were stained with lead citrate followed by uranyl acetate and observed with the transmission electron microscope JEM-1400 (JEOL, Tokyo, Japan).

### 2.7. Measurement of Alcocytes Survival

The investigation of alcocytes survival in vivo was carried out using Swiss mice weighting 25–30 g. All manipulations with the animals were performed in accordance with the international recommendation [26]. Alcocytes from the mouse blood were prepared using the method describer above for human erythrocytes.

The washed alcocytes were labeled with fluorescein isothiocyanate (FITC) according to the method described in [24] (cell suspension with hematocrit of 20%, 10 *µ*M FITC, 4°C, incubation for 30 min), washed for removal of unbound FITC, and resuspended (1 : 1) in 0.9% NaCl. This suspension was administered in the caudal tail vein of the mice at a dose of 0.4 ml/25 g of body mass. At different times (from 5 min to 48 h) blood from the retroorbital plexus was collected. After washing and resuspending to a hematocrit of 1-2%, the percentage of FITC-labeled cells was measured by flow cytometry using the Partec PAS-III particle analyzing system (Partec GMbH) with argon laser (488 nm). The dot-plots of FITC fluorescence versus forward angle light scattering were used.

### 2.8. Enzyme Assays

The following samples were used in activity assays for ADH and ALD: (i) fresh RBC suspensions with added ADH and ALD enzymes; (ii) suspensions of sealed cells; (iii) the supernatant and sediment obtained by centrifugation of the sealed cell suspensions; and (iv) sealed cells after the triple washing procedure. Enzyme activities were determined spectrophotometrically in the solutions or cell lysates using spectrophotometer AMINCO-Bowman Series 2 (Madison, WI, USA) and Multiskan Ascent photometric Plate Reader (Thermo Electron Scientific Instruments, Madison, WI, USA). The initial rates of NAD^+^ reduction were measured at *λ* 340 nm and temperature 25°C.

Assay of ADH was performed in the reaction mixture containing phosphate-hydrazine-glycine buffer (62.5 mM Na_2_HPO_4_, 62.5 mM hydrazine hydrochloride, 17.5 mM glycine, and 8.3 mM EDTA, рН 8.7), 1.8 mM NAD^+^, 0.58 M ethanol, and a sample.

An assay of ALD was performed according to [27] with minor modification using reagents from Sigma (St. Louis, MO, USA). The reaction mixture contained 50 mM sodium pyrophosphate buffer (pH 8.8), 0.6 mM NAD^+^, 5 mM acetaldehyde, and a sample.

The yield of encapsulation for ADH and ALD was calculated as a proportion (in %) of the units of catalytic activity into loaded cells (in katals, corrected for the volume of cells obtained) relative to the enzyme activity added to the RBC suspensions before hypoosmotic dialysis (in katals, corrected for the volume of suspensions obtained).

### 2.9. Measurements of Hemolysis of Alcocytes during Storage

Osmotic resistance of RBCs and alcocytes loaded with ADH and ALD at various levels of osmotic pressure of the dialysis solution (120, 90, and 60 mOsmol/kg) during the second step of dialysis was measured using the release of hemoglobin from the cells. The CPDA-1 solution was added to suspensions of washed cells at a ratio of volumes of 1 : 8 and incubated at 4°C for 7 days. Aliquots were withdrawn on days 1, 3, 4, 5, and 7 and were tested for hematocrit (Hct_susp_) and total hemoglobin after hemolysis of the sample (using absorbance at 405 nm (*A*_susp_)). Then, the suspensions were centrifuged at 1000*g* for 5 min at 4°C. The absorbance of hemoglobin in the supernatant was measured at 405 nm (*A*_supern_). Hemolysis (in %) was calculated as follows:

(1) $$\begin{array}{c} hemolysis=\left(\;100\%-{Hct}_{susp}\;\right)\cdot \frac{A_{supern}}{A_{susp}}. \end{array}$$

### 2.10. Ethanol and Pyruvate Assays

Alcocytes were incubated at 37°С in a buffer containing 50 mM HEPES, 5 mM MgSO_4_, 110 mM NaCl, 3 mM KCl, 1.2 mM NaH_2_PO_4_, 2 mM CaCl_2_, and 10 mM glucose (pH 7.5). In some experiments, the media were supplemented with pyruvate, NAD^+^, and NADH. Aliquots were withdrawn at different time points and ice-cold 10% HClO_4_ was added at a ratio of 1 : 1 (v/v). The samples were kept in an ice bath for 15 min and then centrifuged at 4100*g* for 3 min. The acid extract was neutralized with saturated K_2_CO_3_ and, after 10 min, centrifuged. The second supernatant was frozen and stored at –80°C until assay for ethanol [28].

The ethanol concentration was measured by oxidation of ethanol to acetaldehyde in the presence of ADH and NAD^+^, and NADH generation was measured at 340 nm. The reaction mixture for the ethanol assay contained phosphate-hydrazine-glycine buffer (see above), 1.8 mM NAD^+^, 33.3 *µ*katal/ml of ADH, and the neutralized extract.

Pyruvate was assayed enzymatically by a reduction of pyruvate to lactate in the presence of LDH and NADH [28]. The oxidation of NADH was monitored at 340 nm using a recording spectrophotometer (AMINCO-Bowman Series 2, Madison, WI, USA). The reaction mixture for the pyruvate assay contained 62.5 mM sodium phosphate buffer (рН 7.5), 0.2 mM NADH, 25 *µ*l of acid extract, and 4 U of LDH in a total volume of 0.4 ml. As there is no standard for pyruvate, the results were calculated while considering the molecular extinction coefficient of NADH (6.22 mM^–1^·cm^–1^).

Low steady-state concentration of pyruvate was maintained using prolonged continuous injection of 100 mM pyruvate into the cell incubation mixture. The rate of pyruvate injection was adjusted empirically with measuring pyruvate concentration in the cell suspension for 5 h. The suspension volume was 10 ml, and the maximal amount of pyruvate solution added was no more than 1.5 ml. The rate of pyruvate injection was 0.2–0.3 ml/h providing a pyruvate concentration of 100 *μ*M with of 20% accuracy.

### 2.11. Activities of RBC-Encapsulated Enzymes during Storage

Loaded alcocytes were added to a modified CPDA-1 solution at a ratio 8 : 1 (v/v). The suspensions were stored at 4°C for 7 days. Aliquots were withdrawn on days 1, 4, and 7. Then cells were isolated by centrifugation at 1000*g* for 7 min, washed in three volumes of PBS, and assayed for ADH and ALD activities. The percent ratio of the enzyme activity in each sample to its activity measured immediately after loading was determined.

### 2.12. The Metabolic Pathway of Ethanol Utilization and the Mathematical Model

**Figure 1:**
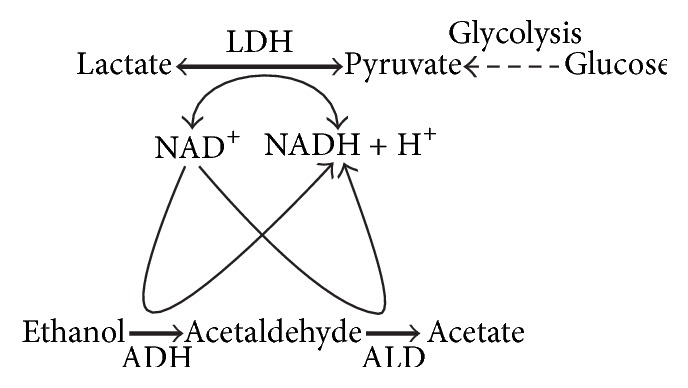
Schematic representation of metabolic pathways of the enzymes encapsulated into RBC for the ethanol detoxification. Alcohol dehydrogenase (ADH) catalyzes an ethanol oxidation to acetaldehyde. After that, aldehyde dehydrogenase (ALD) metabolizes acetaldehyde to acetate. Both reactions involve the NAD^+^ reduction. These enzymes employ glycolysis for their function, since they use lactate dehydrogenase (LDH) of RBC for oxidation of generated NADH to NAD^+^.

Metabolic pathway of ethanol in the alcocytes begins with its oxidation by NAD^+^ catalyzed by ADH (Figure 1). Acetaldehyde, produced in this reaction, oxidizes to acetate with a reduction of one additional molecule of NAD^+^. As a result, complete oxidation of a single ethanol molecule is accompanied by the reduction of two NAD^+^ molecules.

Both, the ADH and ALD reactions use NAD^+^ as coenzyme. If NAD^+^ is present in excess, this coenzyme would not limit the rate of alcohol metabolism. However, the intracellular NAD^+^ concentration in RBCs does not exceed 100 *μ*M [29, 30], while plasma ethanol can achieve levels of 10–100 mM [31, 32]. When ethanol oxidizes by alcocytes, the NAD^+^ concentration presumably decreases and can be insufficient for the effective oxidation of the rest of ethanol. Therefore, ADH and ALD can function under steady-state conditions only when NAD^+^ is constantly regenerated.

Glycolysis is the major metabolic pathway responsible for NAD^+^ maintenance in RBC. The coenzyme reduces in the glyceraldehyde-3-phosphate dehydrogenase reaction, and the resulting NADH oxidizes in the LDH reaction at mean rate of 2−4 mmol per liter of packed RBCs per h (mmol/l_RBCs_·h). As a result, the rates of NAD^+^ reduction and NADH oxidation are equal, so the NAD^+^/NADH ratio is maintained at the homeostatic level. The ADH and ALD reactions compete with glyceraldehyde-3-phosphate dehydrogenase disturbing a balance between NAD^+^ and NADH. Blocking NAD^+^ regeneration should lead to a cessation of the alcohol oxidation after several minutes. LDH is the principal enzyme regenerating NAD^+^ in RBCs, while pyruvate is the main NADH oxidizing substrate. In turn, glycolysis and transport of pyruvate from plasma through the cell membrane are sources of intracellular pyruvate needed for NADH oxidation.

To simulate competition for NAD^+^ between glycolysis and an alcohol oxidation, we developed the simplest model of ethanol oxidation in alcocytes, which consisted of a system of three differential equations. The model included descriptions of changes in the concentrations of three metabolites, NAD^+^, ethanol, and pyruvate (see Results and Discussion), and used the earlier published mathematical model of the erythrocytes, which included all the reactions of glycolysis, adenylates metabolism, ionic balance, and osmotic regulation of erythrocyte volume [21]. Transport of pyruvate from plasma (or buffer in vitro) into RBC was simulated as a constant pyruvate influx *V*_in_.

This model was used to test the sensitivity of ethanol oxidation toward pyruvate availability and NAD^+^ concentration. Additionally, the model predicted the behavior of the metabolic system at saturating concentrations of pyruvate and NAD^+^.

The equations were solved with the DBSolve software package [33].

### 2.13. Statistical Analysis

The results are expressed as the mean ± SEM. Statistical analysis was performed with the OriginPro 8 software (OriginLab Corporation, Northampton, MA, USA). The significance of differences between multiple (more than two) groups was tested by one-way analysis of variance (ANOVA) with Bonferroni correction. Before the analysis, we tested the numerical data for normality of distribution using the Kolmogorov-Smirnov statistics.

## 3. Results and Discussion

In mammals, ADH and ALD localize to the liver and are absent or negligible in RBCs [34]. Their encapsulation and the consequent intravenous administration of enzyme-loaded RBCs to mice lead to increased rates of blood ethanol elimination [12]. However, these rates (maximum 4 mmol/l_RBCs_·h) were much lower than those expected from the activities of encapsulated enzymes (up to 50 mmol/l_RBCs_·h) [13]. Mouse RBCs, loaded with ADH and ALD, were capable of decreasing ethanol levels in the incubation medium (1.7 mmol/l_RBCs_·h) [14]. The reasons for the inadequacy of the data were unclear and not discussed in the literature. One explanation for this discrepancy could be related to the method of enzyme encapsulation, which did not allow the new metabolic pathway to reach adequate activity. The other explanation may be related to a deficiency in the required metabolites. Therefore, the main objectives of our study were (1) to develop a mathematical model of alcohol oxidation using the enzymatic system of ADH and ALD, loaded into erythrocytes, (2) to develop an effective method for the encapsulation of ADH and ALD into RBCs from healthy donors taking into account the model predictions, (3) to analyze the rate-limiting factors of ethanol oxidation into alcocytes experimentally and using developed model, and (4) to examine the efficiency and the other properties of obtained alcocytes experimentally.

### 3.1. Mathematical Modeling of Ethanol Oxidation by Alcocytes

The process of ethanol oxidation into erythrocytes containing encapsulated ADH and ALD can be described by the following set of equations (see Figure 1):

(2) dAlcdt=−VADH;dAlddt=VADH−VALD;dAcetdt=−VALD;dNADdt=−VADH−VALD+VLDH;dNADHdt=VADH+VALD−VLDH;dPyrdt=−VLDH+Vin;dLacdt=VLDH,

where Alc, Ald, and Acet are the concentrations of ethanol, acetaldehyde, and acetic acid, respectively, and *V*_ADH_, *V*_ALD_,* and V*_LDH_ are the rates of the ADH, ALD, and LDH reactions, respectively. The *V*_in_ value reflects the external source of pyruvate, that is, either glycolysis (the pyruvate influx in the experiment with alcocytes) or pyruvate transport through the cell membrane under steady-state plasma pyruvate concentration.

The system uses the following integrals:

(3) $$\begin{array}{c} NAD+NADH={NAD}_{0}, \end{array}$$

Pyr + Lac = Pyr_0_ (this sum is constant only in a calculation regarding the competition between ethanol metabolism and glycolysis),

(4) Alc+Ald+Acet=Alc0,2Alc+Ald−Pyr−NAD=C0.

Thus, we obtain a system with three unknown variables and three initial parameters (initial concentrations):

(5) $$\begin{array}{c} \frac{aNAD}{dt}=-V_{ADH}-V_{ALD}+V_{LDH};\\ \frac{dAlc}{dt}=-V_{ADH};\\ \frac{dPyr}{dt}=-V_{LDH}+V_{in};\\ {\left[\;Alc\;\right]}_{0},{\left[\;NAD\;\right]}_{0},{\left[\;Pyr\;\right]}_{0}. \end{array}$$

The kinetic constants of ADH [15, 16], ALD [17–20], and LDH [21, 22] were taken from the literature (Table 1).

In our calculation, *V*_ADH_, *V*_ALD_, and *V*_LDH_ followed the formulas based on kinetic constants (all* k*_*i*_s are now* K*_*i*_s; the uppercase letters are substituted for the lowercase letters, (see Table 1)):

 
*⁡V*_ADH_ = *V*_1_ = (*V*_11_*∗X*[1]*∗X*[2]/*K*_15_/*K*_12_ − *V*_12_*∗X*[4]*∗*NADH/*K*_13_/*K*_18_)/(1 + *X*[1]/*K*_15_ + *X*[2]*∗K*_11_/*K*_15_/*K*_12_ + *X*[4]*∗K*_14_/*K*_18_/*K*_13_ + NADH/*K*_18_ + *X*[1]*∗X*[2]/*K*_15_/*K*_12_ + *X*[1]*∗X*[4]*∗K*_14_/*K*_15_/*K*_13_/*K*_18_ + *X*[2]*∗*NADH*∗K*_11_/*K*_15_/*K*_12_/*K*_18_ + *X*[4]*∗*NADH/*K*_13_/*K*_18_ + *X*[1]*∗X*[2]*∗X*[4]/*K*_15_/*K*_12_/*K*_17_ + *X*[2]*∗X*[4]*∗*NADH/*K*_16_/*K*_13_/*K*_18_);
 
*⁡V*_ALD_ = *V*_2_ = *V*_21_*∗X*[1]*∗X*[4]/(*X*[1]*∗X*[4] + *X*[1]*∗K*_22_ + (*X*[4]*∗K*_21_ + *K*_25_*∗K*_22_)*∗*(1 + NADH/*K*_28_));
 
*⁡V*_LDH_ = *V*_3_ = (*V*_31_*∗*NADH*∗X*[3]/*K*_35_/*K*_32_–*V*_32_*∗*lact*∗X*[1]/*K*_33_/*K*_38_)/(1 + NADH/*K*_35_ + *X*[3]*∗K*_31_/*K*_35_/*K*_32_ + NADH*∗K*_34_/*K*_38_/*K*_33_ + *X*[1]/*K*_38_ + NADH*∗X*[3]/*K*_35_/*K*_32_ + NADH*∗*lact*∗K*_34_/*K*_35_/*K*_33_/*K*_38_ + *X*[3]*∗X*[1]*∗K*_31_/*K*_35_/*K*_32_/*K*_38_ + lact*∗X*[1]/*K*_33_/*K*_38_ + lact*∗X*[3]*∗*NADH/*K*_35_/*K*_32_/*K*_37_+*X*[3]*∗*lact*∗X*[1]/*K*_36_/*K*_33_/*K*_38_),

where *X*[1] = NAD^+^, *X*[2] = ethanol, *X*[3] = pyruvate, *X*[4] = acetaldehyde, NADH = NAD^+^_0_–*X*[1]; lact = Pyruvate_0_–*X*[3]; *X*[4] = *C*_0_ + *X*[1] + *X*[3]–2*X*[2].

Thus, given the initial concentrations of ethanol, coenzyme NAD^+^, and pyruvate, as well as the initial activities of the enzymes, we can calculate the concentrations of all the metabolites at subsequent time points.

### 3.2. Encapsulation of Enzymes in RBCs

Preparation of the RBC carriers using hypoosmotic dialysis exerted minimal effects on cells. The biochemical, biophysical, and immune properties of loaded RBCs remained similar to those of the native cells. According to [35, 36], cells loaded with enzymes using hypoosmotic dialysis had a lifespan comparable with the lifespan of normal cells in vivo. Therefore, we used hypoosmotic dialysis for obtaining alcocytes.

**Figure 2:**
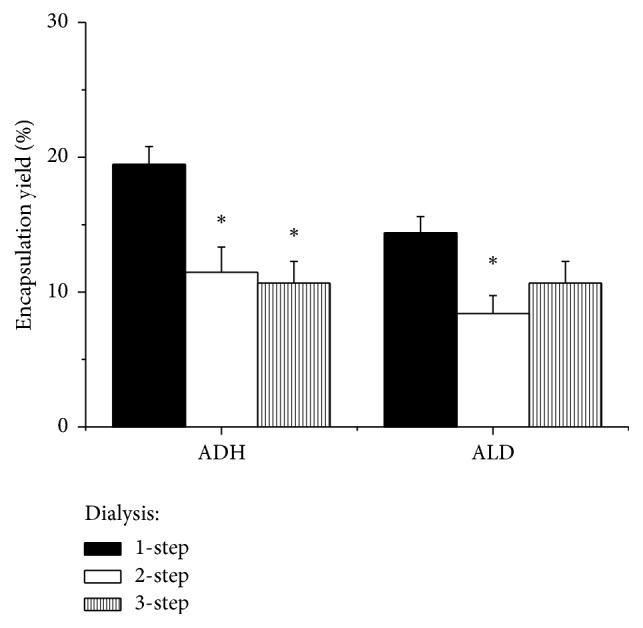
Encapsulation of ADH and ALD in RBCs. The RBC suspension was dialyzed using one- (the first column), two- (the second column), and three-step dialysis (the third column). Values presented are mean ± SEM of 5 to 13 preparations. ^*∗*^The difference is significant compared with one-step dialysis (one-way ANOVA,* P* < 0.05).

To produce the maximum number of enzyme-loaded cells with the maximum encapsulation yield of ADH and ALD, we compared alcocytes obtained using one-, two-, and three-step dialysis. The results presented in Figure 2 demonstrate that the highest encapsulation yield of both ADH (23.0 ± 1.1%) and ALD (16.9 ± 1.3%) and the highest cell recovery (73.5 ± 4.4%) were achieved in one-step dialysis.

Similar values were reported by Lizano et al. [13] with mouse RBCs loaded with ADH and ALD using an electroporation procedure. In two- and three-step dialyses, the cell recovery was 51.2 ± 1.8 and 25.3 ± 2.6%, respectively.

For two-step dialysis, we also examined the dependence of encapsulation of ADH and ALD on the medium osmolality at the second dialysis step (when microruptures in the RBC membrane could be formed). Encapsulation of the two enzymes appeared to be independent of osmotic pressure of the dialysis solution from 56 to 118 mOsmol/kg.

### 3.3. Investigation of the Properties of Alcocytes, Obtained Using Hypoosmotic Dialysis

#### 3.3.1. Changes of RBCs Indexes after Hypoosmotic Dialysis

Hypotonic dialysis caused a decrease in the indexes of alcocytes compared with those of native cells: MCV from 86.2 ± 1.4 to 71.3−77.4 fl, MCH from 29.5 ± 0.7 to 17−20 pg, and MCHC from 34.3 ± 0.6 to 24−26 g/dl (*P* < 0.05), respectively. The MCH and MCHC of alcocytes were decreased, probably due to a leakage of hemoglobin from cells during dialysis. Similar decreases in MCV and MCH were described in the literature [37, 38]. MCV, MCH, and MCHC of alcocytes were virtually independent of the number of dialysis steps and of osmolality of the dialysis solution at the second dialysis step.

#### 3.3.2. Osmotic Resistance of Alcocytes

**Figure 3:**
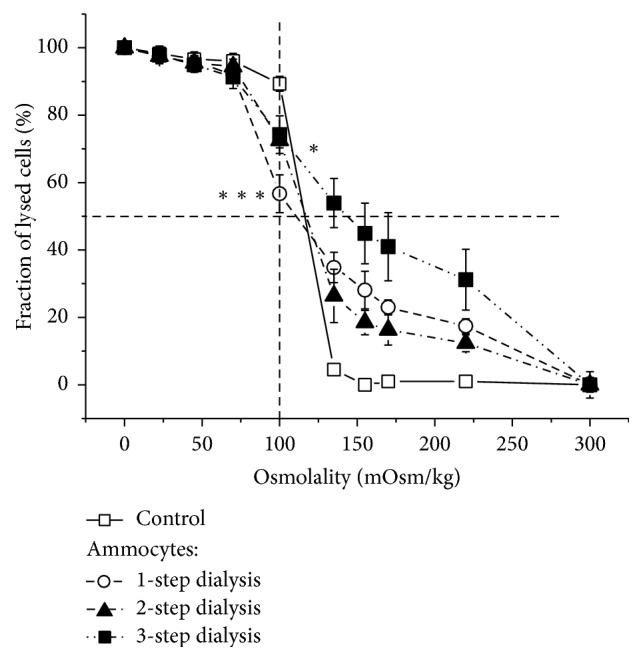
Osmotic stability of alcocytes. Curves of osmotic resistance of control RBCs (□) and alcocytes prepared using one-step (○), two-step (▲), and three-step (■) dialysis. Results are the mean ± SEM (*n* = 3). The differences in a portion of the lysed cells at 100 mOsmol/kg are significant for ammocytes compared to control with ^*∗*^*P* < 0.05 (for 2- and 3-step dialysis), or ^*∗∗∗*^*P* < 0.001 (for 1-step dialysis) (one-way ANOVA with Bonferroni correction).

The investigation of osmotic resistance of alcocytes obtained using hypoosmotic dialysis with the different numbers of steps showed that the curves of osmotic resistance for alcocytes and native RBCs differed significantly (Figure 3). The hemolysis of alcocytes began at an osmolality of 220 mOsmol/kg, whereas control cells were resistant to this osmotic pressure. In contrast, at an osmolality of 100 mOsmol/kg, alcocytes were more resistant than control cells, which were nearly fully lysed.

Despite the low number of experiments in Figure 3 (*n* = 3 for each cell type), the statistical treatment of these results revealed significant differences between control erythrocytes and alcocytes concerning fractions of lysed cells (at 100 mOsmol/kg *P* < 0.001 for alcocytes after a one-step dialysis and *P* < 0.05 for alcocytes after two- or three-step dialysis). Other experiments (*n* > 20), performed under similar but not totally identical conditions, showed that alcocytes were always more resistant to low osmotic pressure relative to the control erythrocytes. We suggest that increased resistance of alcocytes to low osmolality can be accounted for by a destruction of the less resistant alcocytes during their preparation and washing and by the lower content of total protein in enzyme-loaded cells, compared with native erythrocytes (by 10−29%, [24]).

A comparison of osmotic resistance curves for alcocytes, obtained with different numbers of dialysis steps, showed that the cells after three-step dialysis were the least resistant. The Wc_50_ values were 117, 110, 117, and 144 mOsmol/kg for native cells and alcocytes obtained using one-, two-, and three-step dialysis, respectively. These parameters were similar for native RBCs and alcocytes, derived using one- and two-step dialysis.

#### 3.3.3. Stability of Alcocytes

**Figure 4:**
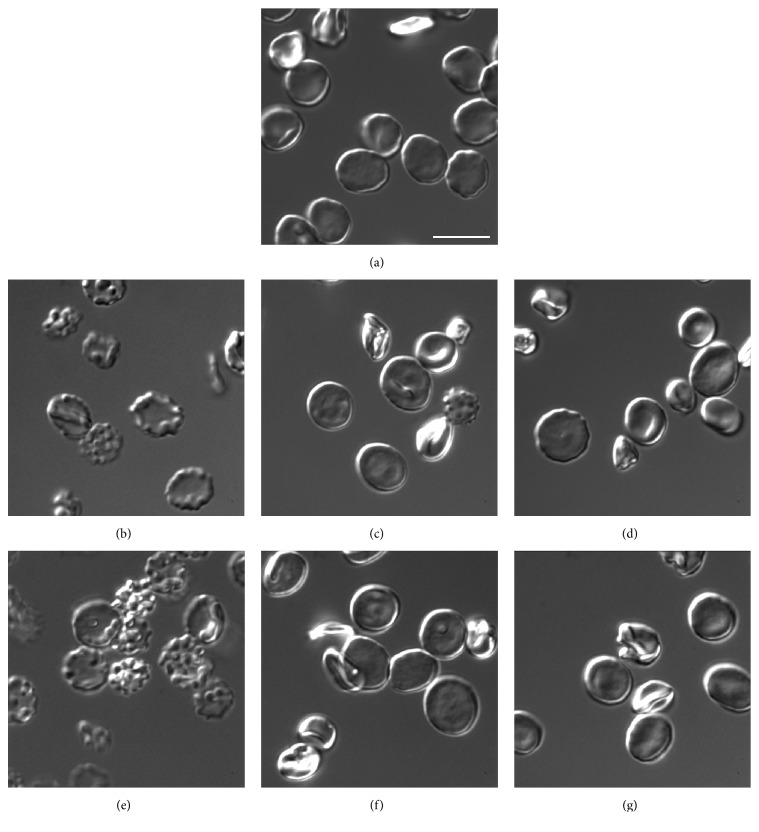
Images obtained by differential interference contrast (DIC) microscopy for initial erythrocytes and erythrocytes after hypoosmotic dialysis with subsequent sealing of the cells in isotonic medium. Initial native erythrocytes (a). Erythrocytes after procedures of hypoosmotic dialysis, sealing, and additional 2 h relaxation of sealed cells (37°C) carried out in the absence (b, c, d) or presence (e, f, g) of ADH (10 IU/ml of RBCs suspension), respectively. The scale bar corresponds to 10 *µ*m. The cells were fixed by 2.5% glutaraldehyde in PBS. Microscope: Zeiss Cell Observer Z.1, objective 100x 1.3 NA, camera QuantEm 512sc. Magnification of 1600x.

Stability of the RBCs uploaded by ADH and ALD was investigated using a microscope, as well as by measuring their survival in vivo. Changes in the erythrocyte form occurring immediately after a one-step hypotonic dialysis were discovered with differential interference contrast (DIC) microscopy (Figure 4). Most parts of the cells restored their form after an isotonic sealing procedure (Figures 4(c) and 4(f)), but a small part of the cells retained the changed form. Only a minor further restoration of these changes was observed after 2-3 h of additional cell relaxation at temperature of 37°C (Figures 4(d) and 4(g)).

**Figure 5:**
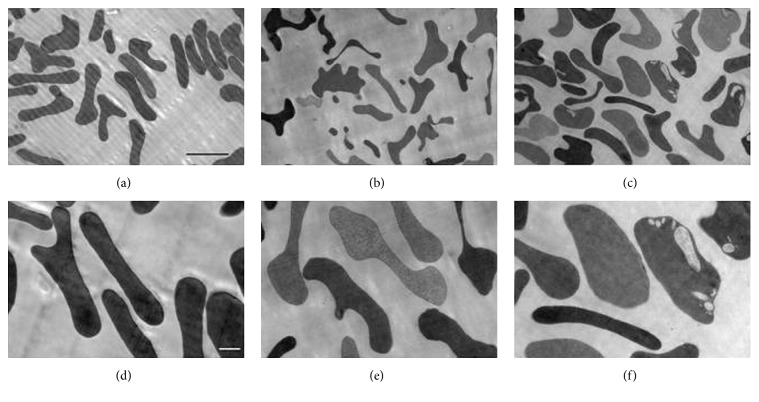
Images obtained by transmission electron microscopy for RBCs at different steps of ADH encapsulation by the method of hypoosmotic dialysis/isoosmotic resealing. Activity ADH is equal to 10 IU/ml of RBC suspension. (a, d) Initial native erythrocytes. (b, e) The RBCs immediately after a hypoosmotic dialysis procedure. (c, f) Loaded RBCs after resealing and additional 2 h cells relaxation at 37°C. Magnification corresponds to 2000x (a, b, c) or 5000x (d, e, f). The scale bars correspond to 5 *µ*m (a, b, c) and 1 *µ*m (d, e, f).

The images of the RBCs at different steps of hypoosmotic dialysis/isoosmotic resealing procedure obtained using transmission electron microscopy (Figure 5) confirmed these conclusions and showed additionally that after resealing new areas appeared inside some RBCs (Figure 5(f)).

**Figure 6:**
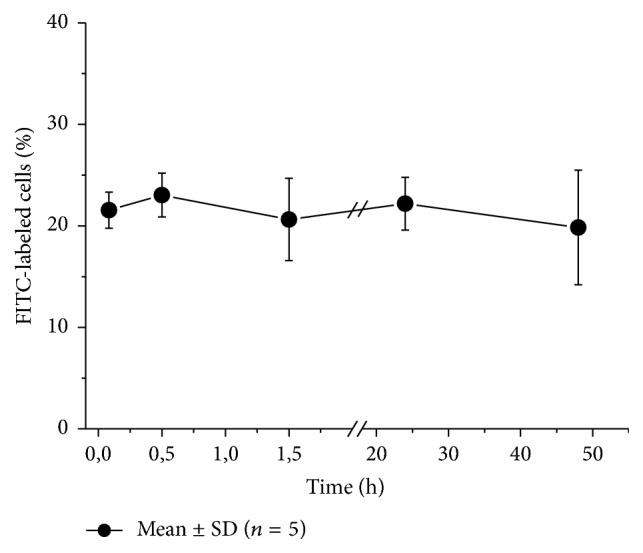
Percentage of FITC-labeled alcocytes from the total number of RBCs in mice in different times after transfusion. Mean values and standard deviations are presented (*n* = 5). The percentage of labeled cells in the initial alcocyte suspension was on average 98.56%. Each mouse (25 g) obtained 0.4 ml of alcocyte suspension (Ht 50%). The first point in the figure corresponds to 5 min after transfusion. The decrease in the proportion of labeled cells in comparison to the initial labeled suspension corresponds to the dilution of this suspension during in vivo administration.

Stability of mouse alcocytes obtained with hypoosmotic dialysis method was proved also by the fact that they had good survival in vivo after transfusion to the animals (Figure 6). In these experiments the alcocytes were labeled with fluorescein isothiocyanate (FITC). The percentage of labeled cells from the total number of erythrocytes was measured by flow cytometry at different times after transfusion. In this study the period of observation was only 48 h, but the percentage of labeled cells did not decrease over this time. On the other hand, the measurements carried out before with glutamine synthetase loaded RBCs showed very good survival of these cells over 8 days (data is not presented).

#### 3.3.4. Hemolysis of Alcocytes and Changes of the Encapsulated Enzymes Activities during Storage

Hemolysis of alcocytes during their storage at 4°C did not depend on the dialysis solution osmolality at the second dialysis step and was 10−15% higher than that of native RBCs.

We suppose that RBCs, loaded with ADH and ALD, should be long-circulating, rapidly acting bioreactors. Therefore, it was important to test the ability of enzymes in alcocytes to retain the activities, as an index of their functional efficiency. This ability for each of two enzymes was estimated during the period of alcocytes storage. The results showed that ADH was more stable than ALD, since enzyme activities decreased by approximately 12% and 48%, respectively, over 7 days. These results are consistent with data reported by others [13, 39].

The data presented above demonstrated that the one-step procedure of hypotonic dialysis was the optimal method for encapsulating ADH and ALD in RBCs according to cell recovery, encapsulation yield, osmotic resistance, and RBC-indexes. Thus, this method was applied in further experiments.

### 3.4. Determination of the Rate-Limiting Factors in Ethanol Oxidation by Alcocytes

#### 3.4.1. Rate of Ethanol Oxidation Depends on the Presence of Pyruvate

Not enough NAD^+^ arises in the LDH reaction to maintain effective ethanol oxidation, since a pyruvate concentration in the blood is low (53.3 ± 21.5 *µ*M) [28]. In the RBC, pyruvate is generated by glycolysis with a maximum rate of 3-4 mmol/l_RBCs_·h.

One molecule of NAD^+^ is required to run glyceraldehyde 3-phosphate dehydrogenase in glycolysis. In addition, two more molecules are needed for oxidation of glucose with a formation of two molecules of pyruvate. On the other hand, oxidation of two formed NADH molecules requires two molecules of pyruvate.

Model calculation showed that some quantity of alcohol could be oxidized by alcocytes. It was suggested that ADH and ALD could compete with glyceraldehyde 3-phosphate dehydrogenase for NAD^+^. The upper limit of ethanol oxidation rate could not be higher than half of the glycolysis rate, that is, 1.5–2 mmol/l_RBCs_·h. This suggests that the inefficiency of alcocytes in ethanol elimination could be accounted for by the low rates of pyruvate generation in glycolysis, as well as by low rates of its influx from plasma.

The LDH enzyme is responsible for this process in the RBC. The LDH activity in RBCs is relatively high under optimal conditions (33 ± 1 IU/ml_RBCs_ in native RBCs or 25 ± 3 and 24 ± 2 IU/ml_RBCs_ in erythrocytes after a hypotonic dialysis/isotonic sealing procedure in the absence or presence of the enzyme, resp., [24]). Thus, LDH activity is approximately three orders of magnitude higher than the rate of glycolysis. However, this reaction requires continuous supply of pyruvate to be run. The RBC pyruvate concentration is 30–50 *µ*M [24, 28], which is evidently not enough for providing the LDH reaction with the substrate. Therefore, to work effectively, alcocytes should be supplemented also with pyruvate. Our study was aimed at analyzing the functional efficiency of the enzymes system (ADH + ALD) encapsulated into RBCs at low and high intracellular pyruvate concentrations.

**Figure 7:**
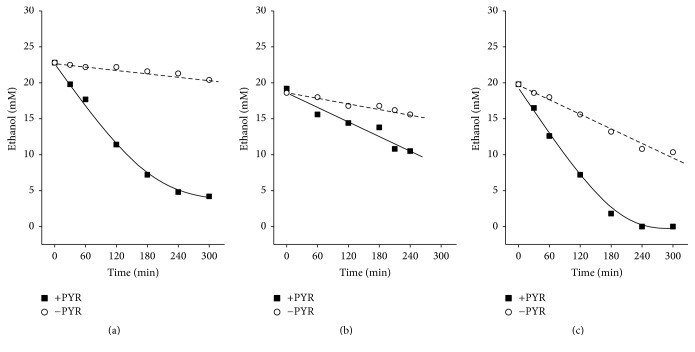
Ethanol oxidation by alcocytes in vitro at various concentrations of pyruvate and NAD^+^. (a) Alcocytes were incubated in the medium containing 60 mM pyruvate (■) or without pyruvate (○). The initial rates of ethanol oxidation in the absence and presence of pyruvate were 1.8 and 30 mmol/l_RBCs_·h, respectively. Parameters of the mathematical model (lines) were as follows: 170 nkatals/ml for ADH, 43 nkatals/ml for ALD, and 173 nkatals/ml for LDH; hematocrit of 28%; and 16 mM glucose. (b) Alcocytes were produced in the absence (○) or presence (■) of NAD^+^ in the outer dialysis circuit. The initial rates of ethanol oxidation were 2.5 ± 0.4 and 7.3 ± 1.2 mmol/l_RBCs_·h, in the absence and presence of NAD^+^, respectively. Parameters of the mathematical model (lines) were as follows: 150−167 nkatals/ml for ADH, 25−33 nkatals/ml for ALD, and 100 nkatals/ml for LDH; hematocrit of 25−27%; and 43 *µ*M NAD^+^ (■) or 9.5 *µ*M NAD^+^ (○). (c) Alcocytes were produced in the presence of 100 *µ*M NAD^+^ (○) or 100 *µ*M NAD^+^ plus 100 *µ*M NADH (■) in the outer dialysis circuit. Initial rates of ethanol oxidation were 10.4 ± 1.2 and 37.4 ± 0.4 mmol/l_RBCs_·h, respectively. Parameters of the mathematical model were as follows: 150 nkatals/ml for ADH, 25 nkatals/ml for ALD, 100 nkatals/ml for LDH, and hematocrit of 25–27%. Typical experiments are presented.

To test the hypothesis that ethanol oxidation is limited by the glycolytic pyruvate generation, we incubated alcocytes either with or without different concentrations of exogenous pyruvate (up to 60 mM) for 5 h. Aliquots of the incubation mixture were taken at time intervals of 30−60 min and used for ethanol determination. As shown in Figure 7(a), the rate of ethanol oxidation by alcocytes in the absence of exogenous pyruvate was 1.8 mmol/l_RBCs_·h, which was consistent with the rate of RBC glycolysis and results given by Lizano et al. [13]. At 60 mM pyruvate, the initial rate of ethanol oxidation by alcocytes was 30 ± 2.7 mmol/l_RBCs_·h (Figure 7(a)), 17 times higher than that without added pyruvate. Additionally, the addition of NAD^+^ or NAD^+^ + NADH to a dialysis solution resulted in an increased rate of ethanol elimination using obtained alcocytes (Figures 7(b) and 7(c), resp.).

The results obtained suggested that exogenous pyruvate was essential for effective function of alcocytes. The results also supported the proposition that the rate of glycolysis was insufficient to generate and maintain the necessary concentration of pyruvate.

**Figure 8:**
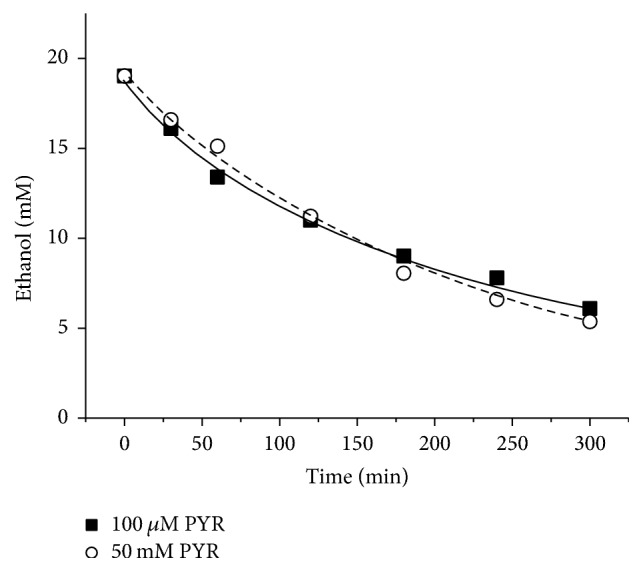
Changes in the ethanol concentration during incubation of alcocytes at different pyruvate concentrations. (■) Pyruvate concentration was maintained at 100 *µ*M; (○) initial pyruvate concentration was 50 mM (as well as 10 mM, or 1 mM, not shown). At 100 *µ*M and 50 mM pyruvate, the initial rate of ethanol oxidation was about 30 mmol/l_RBCs_·h. Typical experiments are presented. In the mathematical model (lines), ADH activity of 83 nkatals/ml, ALD activity of 33 nkatals/ml, LDH activity of 225 nkatals/ml, and hematocrit of 21% were used.

There have been no previous studies in the literature indicating that the concentration of pyruvate could be sufficient for effective ethanol oxidation using alcocytes. In the present study, the mathematical modeling showed that the high rate of ethanol oxidation by alcocytes could be achieved using either the initially high or low, but permanent concentrations of pyruvate. To test this prediction of the model, we incubated alcocytes in vitro in pyruvate-rich and pyruvate-poor media. In the former case, suspensions initially contained 50 mM pyruvate, which was 2.5 times higher than the initial concentration of ethanol. In the latter case, the 100 *µ*M concentration of pyruvate remained more-or-less constant throughout the 5 h of incubation (in contrast to the experiment in Figure 7(a) without pyruvate). A constant supply of pyruvate for RBC suspensions and maintenance of its steady-state concentration were provided with a micropump. The rate of ethanol elimination at a constant 100 *µ*M pyruvate concentration did not differ from that at the initially excessive pyruvate concentration (Figure 8); both were equal to approximately 30 mmol/l_RBCs_·h. This suggests that a constant pyruvate concentration of 100 *µ*M is sufficient for the optimal rate of NADH oxidation and effective functioning of the ADH + ALD enzyme system.

#### 3.4.2. Compensation of a NAD^+^ Loss Increases the Rate of Ethanol Oxidation

Limited availability of the cofactor NAD^+^ for the ADH and ALD reactions could be another cause of disagreement between the low rate of ethanol oxidation and high activities of RBC-encapsulated enzymes. Earlier Lizano et al. [12] proposed that ethanol oxidation by RBCs loaded with ADH and ALD could be limited by the availability of NAD^+^, but this proposition was not tested experimentally. Moreover, these authors added only 5 *µ*M NAD^+^ to the reaction mixture in vitro containing 30 mM ethanol and did not add NAD^+^ to the RBC suspensions of mice, which were administered to the animals, limiting ethanol oxidation in an artificial manner both in vitro and in vivo. Therefore, the third objective of our study was to investigate the functional efficiency of the ADH + ALD enzyme system at the low and high intracellular NAD^+^ concentrations.

A decrease in the intracellular concentration of free NAD^+^ in alcocytes, resulting from its reduction to NADH, can gradually decrease the rate of ethanol oxidation. NAD^+^ could also be lost from cells during hypoosmotic dialysis when pores form in the cell membrane. To examine this hypothesis and to compensate for the loss of NAD^+^ from cells, the experiments were carried out with alcocytes, which were prepared using the dialysis solution containing 100 *µ*M NAD^+^. In one group, a coenzyme was also added to the suspension of erythrocytes before resealing. The rate of ethanol elimination by the cells prepared in the presence of NAD^+^ in the dialysis solution was appreciably higher than that in the absence of NAD^+^ (Figure 7(b)).

Excess NAD^+^ can lead to an imbalance in the cytosolic NAD^+^/NADH redox system. The cytosolic ratio for free NAD^+^/NADH in the erythrocytes is 471 ± 143 as estimated using the indirect method [40]. When the total nucleotides (free plus protein-bound) were directly measured, a physiological ratio NAD^+^/NADH in the erythrocytes was equal to 2.2–2.5 [30] but varied from 0.5–0.6 to 20–30 under various conditions [37]. To keep the cytosolic NAD^+^/NADH ratio in alcocytes supplemented with excess NAD^+^, and to compensate for possible loss of NADH from the cells during hypotonic dialysis, we tested how the addition of NADH to the dialysis solution could impact ethanol oxidation using the obtained alcocytes. Alcocytes produced in the presence of both NAD^+^ and NADH in the outer dialysis solution metabolized ethanol much faster than those prepared in the presence of only NAD^+^ (Figure 7(c)). The experimental data presented in Figure 7(c) were used in the mathematical simulation. Applying our model to these data, we calculated the intracellular NAD^+^ concentrations after the addition of the cofactor to the dialysis solution, which were equal to 43.6 ± 4.2 *µ*M, in agreement with NAD^+^ levels in the native RBCs [29, 30]. Mathematical simulation as well as the in vitro experiments demonstrated that, following the replenishment of intracellular NAD^+^ concentration, a 3.6-fold increase in the rate of ethanol oxidation was possible.

#### 3.4.3. Ethanol Oxidation by Alcocytes Is Not Limited by Enzyme Amounts

The hypothesis that functional deficiency of enzymes is a cause of slow ethanol oxidation by alcocytes was tested with mathematical modeling applied to experimental data of [13] at saturated NAD^+^ (1 mM) and pyruvate (100 *µ*M) concentrations. The result of the simulation showed that 30 mM ethanol was oxidized for 7 h, providing an average rate of ethanol oxidation of 17 mmol/l_RBCs_·h, ten times higher than the rate reported in [13]. All the parameters of the model excluding the concentrations of pyruvate and NAD^+^ were similar to those presented in [13]. This result indicates that ethanol oxidation by alcocytes was not limited by encapsulated enzyme activities.

### 3.5. A Comparison of Experimental and Theoretical Data

**Figure 9:**
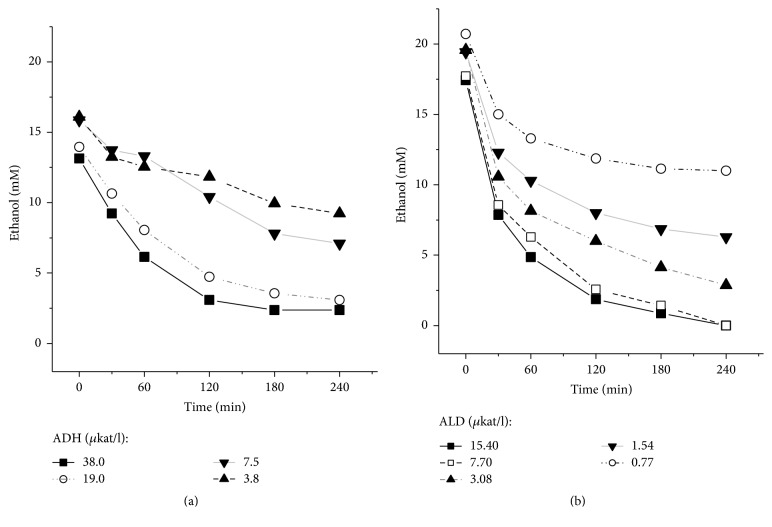
Kinetics of ethanol elimination at different values of the activities of ADH and ALD in the cell-free system. (a) The ADH activity was of 38 *µ*kat/l (■), 19 *µ*kat/l (○), 7.5 *µ*kat/l (▲), and 3.8 *µ*kat/l (▼), and the activities of ALD and LDH were of 7.7 *µ*kat/l and 288 *µ*kat/l, respectively. (b) ALD activity was of 15.4 *µ*kat/l (■), 7.7 *µ*kat/l (□), 3.08 *µ*kat/l (▲), 1.54 *µ*kat/l (▼), and 0.77 *µ*kat/l (○), and the activities of ADH and LDH were of 38 *µ*kat/l and 288 *µ*kat/l, respectively. Typical results are presented, *n* = 3.

We performed a matching examination of model results and experimental data based on experimental in vitro observations in a cell-free system. In addition to ADH and ALD, the incubation buffer also contained LDH to regenerate NAD^+^ from NADH formed during the oxidation of ethanol to acetate. Kinetics of ethanol elimination for the different levels of ADH and ALD activities in the system are shown in Figure 9.

**Figure 10:**
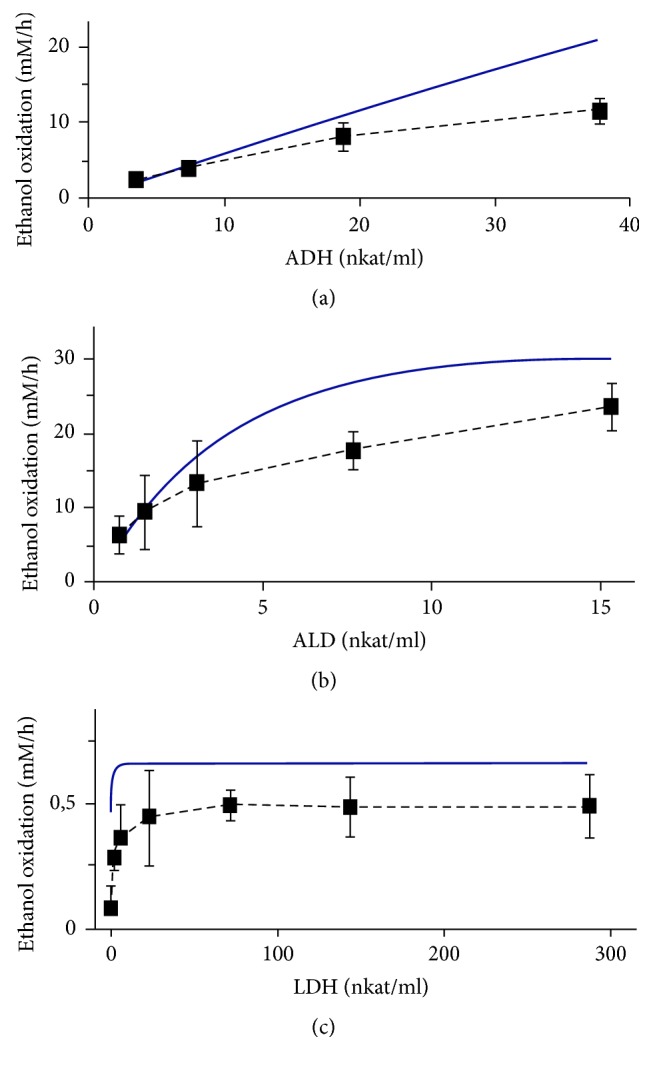
Dependencies of the rate of ethanol oxidation against the activity of the enzymes in a cell-free system. (a) For ADH study the incubation mixture contains ALD of 7.68 *µ*kat/l, LDH of 288 *µ*kat/l, 16−20 mM ethanol, 50 mM pyruvate, and 100 *µ*M NAD^+^. (b) For ALD study the incubation mixture contains ADH of 38 *µ*kat/l, LDH of 288 *µ*kat/l, 16−20 mM ethanol, 50 mM pyruvate, and 100 *µ*M NAD^+^. (c) The LDH activity was studied in the incubation mixture containing ADH of 3.8 *µ*kat/l, ALD of 1.54 *µ*kat/l, 5 mM ethanol, 20 mM pyruvate, and 100 *µ*M NAD^+^. Experimental results (■) are presented as the mean values ± SEM (*n* = 3). Solid lines present the results of the mathematical simulation.

Using experimental results shown in Figure 9, the initial rates of ethanol oxidation were calculated at different concentrations of each enzyme (ADH, ALD, and LDH) and were compared with the calculated rates using mathematical simulation. It is evident from Figure 10 that the experimental data were satisfactorily qualitatively consistent with the results of mathematical modeling.

## 4. Conclusions

The developed mathematical model of ethanol oxidation by erythrocytes containing ADH and ALD, which takes into account the processes of glycolysis in erythrocytes, allows an accurate description of ethanol oxidation by alcocytes. This model is capable of predicting rate-limiting factors of ethanol oxidation in alcocytes. The main of these factors are the low rate of NAD^+^ regeneration and low availability of pyruvate for LDH, but not the activities of the encapsulated enzymes. The procedure of encapsulation for ADH and ALD based on hypoosmotic dialysis was developed. It was shown that the one-step procedure seemed to be optimal. Relative activities of encapsulated ADH and ALD were moderately stable during alcocyte storage, decreasing by no more than 48% over 7 days. Experiments confirmed the model predictions, indicating that the activities of loaded ADH and ALD enzymes did not limit ethanol oxidation by alcocytes. Availabilities of both pyruvate and free NAD^+^ were rate-limiting factors in ethanol oxidation by alcocytes. The rate of ethanol metabolism by alcocytes, loaded with the whole enzyme system of ADH, ALD, and NAD^+^, might be 20 to 40 times as high as that reported in previous studies. In the body, plasma pyruvate can be a source of pyruvate for alcocytes. Despite low plasma pyruvate concentration (approximately 50 *µ*M), this level remains stable due to the pyruvate supply from different cells.

A simple calculation demonstrate that alcocytes with sufficient therapeutic activity can be obtained under the usual conditions for the incorporation of ADH and ALD into erythrocytes. Let us consider a patient weighing 70 kg who has approximately 5 liters of blood (with a hematocrit of 45%) and assume that for the preparation of the cells carriers we can take 500 ml of blood from this patient. After the production of the RBCs loaded with ADH and ALD (using a hypoosmotic dialysis and subsequent isotonic sealing), approximately 100 ml of alcocytes will be obtained (assuming that the cell yield at sealing is ~50%). After transfusion of these erythrocytes to the patient, their Ht will be 4%, that is, approximately 6 times less than that used for the calculation in Figure 7(c). To obtain the rate of alcohol utilization in the patient equal to that simulated in Figure 7(c), dialysis and sealing should be performed in the presence of added NAD^+^ (100 *μ*M) and NADH (100 *μ*M). Given that 1 IU of enzyme activity amounts to 16.67 nkat, it can be calculated that the enzyme activities included in the red blood cells in our case should be approximately as follows:

  ADH: (150/16.67)·6~54 IU/ml of RBCs.
  ALD: (25/16.67)·6~9 IU/ml of RBCs.

Such concentrations of ADH and ALD can easily be incorporated into RBCs. It should be noted that all these calculations are valid with an LDH activity of 100/16.67~6 IU/ml of RBCs (Figure 7(c)). Given that the activity of LDH in erythrocytes is high enough [24], it can be assumed that it will not be a factor limiting the effectiveness of the obtained alcocytes.

Thus, we suggest that, for alcoholic patients with acute intoxication, administration of erythrocytes (from the same patient or from the proper donors), which have been previously loaded with components of the whole metabolic system of ethanol oxidation, may be an improved method for rapid detoxification of blood alcohol and acetaldehyde based on the metabolism of the toxin.

## Figures and Tables

**Table 1:** Kinetic constants for ADH, ALD, and LDH^1^.

Constant^2^	ADH	ALD	LDH
*K*_*a*_, *µ*M	180 (*K*_11_)	20 (*K*_21_)	24 (*K*_31_)
*K*_*b*_, *µ*M	17000 (*K*_12_)	9 (*K*_22_)	120 (*K*_32_)
*K*_*p*_, *µ*M	800 (*K*_13_)		6000 (*K*_33_)
*K*_*q*_, *µ*M	100 (*K*_14_)		100 (*K*_34_)
*K*_*ia*_, *µ*M	270 (*K*_15_)	70 (*K*_25_)	8 (*K*_35_)
*K*_*ib*_, *µ*M	90000 (*K*_16_)		130 (*K*_36_)
*K*_*ip*_, *µ*M	1100 (*K*_17_)		130000 (*K*_37_)
*K*_*iq*_, *µ*M	31 (*K*_18_)	100 (*K*_28_)	100 (*K*_38_)
*V*_*f*_ (Et), s^−1^	40 (*V*_11_)	1 (*V*_21_)	550 (*V*_31_)
*V*_*r*_ (Et), s^−1^	340 (*V*_12_)		60 (*V*_32_)
*K*_eq_, M × 10^12^	22		3.8

^1^ *K*_*a*_, *K*_*b*_, *K*_*p*_, and *K*_*q*_ are Michaelis constants. *K*_*i*_ is the constant of inhibition. *K*_eq_ is the equilibrium constant.

^2^The indexes *a*, *b*, *p*, and *q* denote the following substrates (in the order of their association with and dissociation from the corresponding enzyme): NAD^+^, ethanol, acetaldehyde, and NADH (for ADH [15, 16]); NAD^+^, acetaldehyde, acetate, and NADH (for ALD [17–20]); and NADH, pyruvate, lactate, and NAD^+^ (for LDH [21, 22]), respectively.
